# Racial factors and inpatient outcomes among patients with diabetes hospitalized with foot ulcers and foot infections, 2003-2014

**DOI:** 10.1371/journal.pone.0216832

**Published:** 2019-05-29

**Authors:** Ché Matthew Harris, Aiham Albaeni, Roland J. Thorpe, Keith C. Norris, Marwan S. Abougergi

**Affiliations:** 1 Department of General Internal Medicine, Johns Hopkins School of Medicine, Division of Hospital Medicine Johns Hopkins Bayview Medical Center, Baltimore, Maryland, United States of America; 2 Department of Medicine, University of Central Florida, Ocala, Florida, United States of America; 3 Johns Hopkins Bloomberg School of Public Health, Baltimore, Maryland, United States of America; 4 Department of Internal Medicine, David Geffen School of Medicine, University of California Los Angeles, Los Angeles, California, United States of America; 5 Department of Internal Medicine, Division of Gastroenterology, University of South Carolina School of Medicine, Columbia, South Carolina United States of America; 6 Catalyst Medical Consulting, Simpsonville, SC, United States of America; Medical University Graz, AUSTRIA

## Abstract

**Background:**

In patients with diabetes, foot amputations among Black patients have been historically higher compared with White patients. Using the National Inpatient Sample database, we sought to determine if disparities in foot amputations and resource utilization have improved over time. We hypothesized there would be improvements and reduced differences in foot amputations between the two races as quality of care and access to healthcare has improved.

**Methods and findings:**

Patients over 18 years old with a principal diagnosis of diabetic foot complications and secondary diagnosis of Diabetes Mellitus were selected. We compared the primary outcome of foot amputations between Black and White patients. Adjusted rates, odds ratios (aOR) and trends of foot amputations among Black and White patients were studied. Healthcare utilization was measured via length of hospital stay (LOS). Of 262,924 patients, 18% were Black. Following adjustment for confounders, major foot amputations decreased among Whites (1.5% in 2003 to 1.1% in 2014) and Blacks (2.1% in 2003 to 0.9% in 2014). On pooled analysis, Black patients had higher adjusted odds of major foot amputations in 2003–2004 [aOR 1.7; (1.16–2.57), p<0.01]. Disparities in major foot amputations disappeared in 2013–2014 [aOR: 0.92 (0.58–1.44), p = 0.70]. Black patients had declining but persistently longer LOS (adjusted mean difference (aMD): 1.1 days (0.52–1.6) p<0.01 in 2003–2004 and 0.46 days (0.18–0.73) p<0.01 in 2013–2014). The main limitation of the study was that the NIS uses ICD-9 and ICD-10 CM codes, and hence prone to incorrect or missing codes.

**Conclusions:**

Major foot amputations declined among Black and White patients hospitalized with Diabetic foot complications between 2003 and 2014. The observed difference for amputations in 2003–2004 was absent by 2013–2014. Future research to determine specific contributors for this reduction in health disparities is needed for ongoing improvements and sustainability.

## Introduction

Diabetic foot ulcers and infections contribute to increased morbidity and mortality in affected individuals [1–4]. Foot amputations and premature death are dreaded outcomes of Diabetic foot ulcers and infections; and patients with Diabetes Mellitus who undergo foot amputations are three times more likely to die within a year of the surgery compared with those who have conservative management [5]. Life expectancy for patients with Diabetes Mellitus who undergo foot amputations has been found to be akin to aggressive forms of cancer and end stage congestive heart failure [6,7]. In addition, lower extremity amputations are a significant contributor to inpatient costs associated with managing Diabetic foot ulcers and infections [8–10]. Healthcare costs associated with Diabetic foot ulcers and infections (DFUs/DFIs) have been reported to be as high as $9-$13 billion dollars per year [11].

Racial disparities in frequency of lower extremity amputations have been previously identified, with rates of major amputations being the highest among Black patients [12,13], even after controlling for socioeconomic status [13]. Despite an overall decreasing trend of foot amputation rates over time, the frequency of major amputations was still comparatively higher in Black patients with Diabetes Mellitus insured by Medicare compared with other races [14]. Further, Black patients with Diabetes Mellitus who underwent lower extremity amputations had significantly greater mean total hospitalization charges and mean hospital length of stays (LOS) compared with other races [15]. Despite such disparities in treatment outcomes and resource utilization being a persistent cause for concern [16], no study to date has examined this issue using a national database. We hypothesized that, due to multiple medical and social factors to improve patient care, racial disparities in lower extremity amputations have decreased over the past decade. To test this hypothesis, we examined the trend in rate of major and minor lower extremity amputations among Black and White patients in the United States over the past 12 years (2003 to 2014) using the largest all-payer nationally representative database in the United States.

## Methods

### Database

Data from the years 2003–2014 were obtained from the National Inpatient Sample database. National Inpatient Sample was created by the Agency for Healthcare Research and Quality as part of the Healthcare Cost and Utilization Project, a federal-state-industry partnership [17]. National Inpatient Sample is the largest publicly available national all-payer inpatient care database. It is designed as a stratified probability sample meant to represent all non-federal acute care inpatient hospitalizations in the United States. Briefly, hospitals are stratified according to five major features: ownership/control, bed size, teaching status, urban/rural location, and United States geographical region. An approximate 20% probability sample of all discharges from participating hospitals within each stratum is collected and information about patients’ demographics, diagnoses and resource utilization are entered into the database. Each discharge is weighted to make NIS nationally representative. National Inpatient Sample (NIS) is a discharge level database which contains de-identified clinical and nonclinical data elements, which protects the privacy of patients, physicians, and hospitals as required by data sources and the Health Insurance Portability and Accountability Act. As a result, multiple admissions for a single patient are considered separate discharges and are entered separately in the database. From 2003 to 2014, NIS included between 7 to 8 million discharges yearly from 994 to 4,411 hospitals in 37 to 45 states across the United States [17].

### Study population

Admissions were included in the study if they had a principal diagnosis of foot ulcers/foot infections, and a secondary diagnosis of Diabetes Mellitus. Patients were excluded if they were younger than 18 years of age. The following ICD-9-CM codes were used to identify eligible admissions:

1. Foot ulcers/infections: 707.14 (non-pressure chronic ulcer of heel and mid-foot), 707.15 (ulcers of other part of foot-toes), 681.10 (cellulitis and abscess of toe), 681.11 (onychia and paronychia of the toe), 682.7 (cellulitis and abscess of foot-except toes), 730.07 (acute osteomyelitis of ankle and foot), 730.17 (chronic osteomyelitis of ankle and foot).
2. Diabetes Mellitus: 250.xx

### Study outcomes

The primary outcome was major lower limb amputation, described as a foot amputation above the ankle. Secondary outcomes were: i) minor lower limb amputations (amputations below the ankle), ii) combined major and minor lower limb amputations, and iii) hospital length of stay.

Patient vital status at discharge, patients’ demographics and hospital length of hospital stay is coded directly in NIS. The following ICD -9 -CM codes were used to identify the study outcomes: Above the ankle amputations: 84.13–84.19 and foot amputations: 84.11–84.12.

### Patient and hospital characteristics

The main independent variable was patient race. Race is directly coded in NIS and contains the following categories: Whites, Blacks, Hispanic, Asian, Native American and other. The current study only analyzed White and Black patients. Time in years was used as a continuous predictor.

Multiple patient and hospital level characteristics were collected and adjusted for in the analysis as potential confounders. Those were: 1) patient level variables: age (in years), gender, median household income in the patient’s zip code, insurance and co-morbidities measured using the Charlson comorbidity index [18]; 2) hospital level variables: hospital bed size, teaching status, urban location, and region.

The Johns Hopkins University School of Medicine’s Institutional Review Board deemed the study exempt from approval because it involved retrospective analyses of de-identified data.

### Statistical analyses

Patient demographics, co-morbidities, and hospital characteristics were compared from the years 2003 through 2014 using Pearson’s χ^2^ test for categorical variables and one-way analysis of variance for continuous variables. Adjusted yearly rates of amputations (minor, major and total) were adjusted for the multiple confounders listed above using predictive margins [19].

We calculated adjusted mean differences and adjusted odds ratios using multivariate linear and logistic regression analysis, respectively. Multivariate regression models were built by including all potential confounders that were significantly associated with the outcome on univariate analysis with a p-value cutoff of 0.2. Variables that were deemed important determinants of the outcome based on previous literature were forced into the models. Statistical tests for trends across years were performed for all study outcomes. To calculate the trend p-value, a regression model was built with the outcome of interest as the dependent variable and the year as the independent variable, along with the confounders listed above. Linear and logistic regressions were used for continuous and binary outcomes, respectively. An interaction term (race * year) was added to the regression model to measure the difference in trends across the years between the two racial groups.

We used Stata 15.0 (StataCorp, College Station, TX) to perform all statistical analyses. Analyses accounted for survey design complexity (stratification, clustering, and weighting) based on Healthcare Cost and Utilization Project NIS’ analytic guidelines by incorporating sampling weights, primary sampling units, and strata [17] and produce nationally representative unbiased results, variance estimates and p values. All p values were 2 sided and type I error was set at 0.05.

## Results

### Patient and hospital characteristics

**Fig 1:**
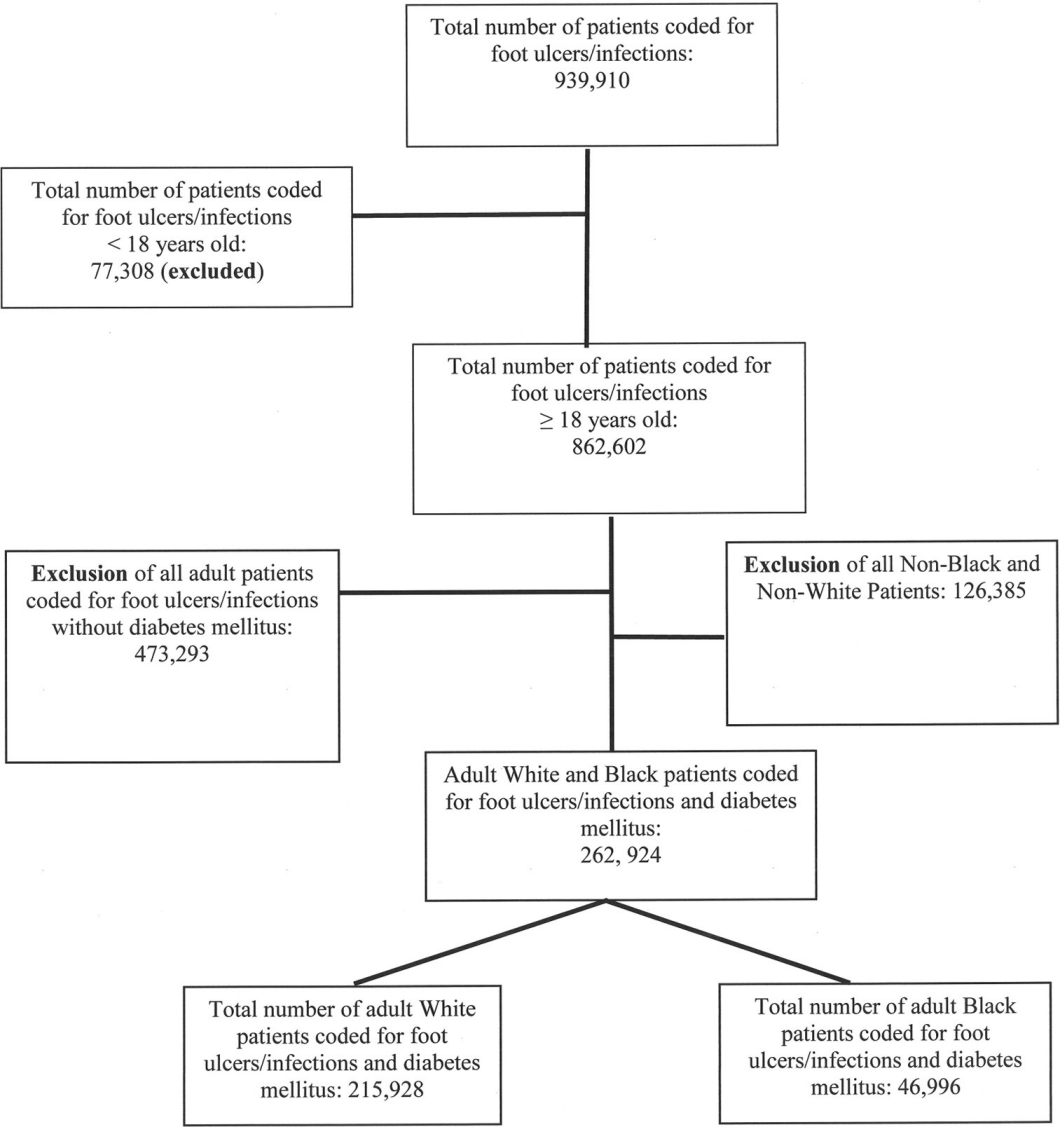
Patients were selected from the 2003–2014 National Inpatient Sample database. Inclusion criteria were foot ulcer or infection, Diabetes Mellitus, and age ≥ 18 years old.

Fig 1 shows the flow diagram for study inclusion between 2003–2014. There were 939,910 hospitalizations for DFUs/DFIs from 2003 to 2014. A total of 262,924 were included in the study. Of those, 215,928 were White, and 46,996 were Black. Compared to White patients, Black patients were significantly younger (mean age: 57.4±0.4 vs. 62.9±0.2 years, p<0.01), more likely to be female (45.5% vs 39.1%, p<0.01), had a greater likelihood to be uninsured (7.9% vs 5.7%, P<0.01) and to belong to the lowest median household income quartiles (54.6% vs 28.3%, p<0.01) (Table 1).

**Table 1:** Demographics and hospital outcomes comparing White and Black patients hospitalized with diabetic foot complications, National Inpatient Sample (2003–2014).

Characteristics/Outcomes	White patients N = 215,928	Black patients N = 46,996	p-value
Patient characteristics			
Age (years)(mean +/-SE)	62.9 +/- 0.2	57.4 +/-0.4	<0.01
Female (%)	84,427 (39.1)	21,383 (45.5)	<0.01
Charlson comorbidity score, n (%)			
0	10,580 (4.9)	2,678 (5.7)	0.008
1	56,141 (26.0)	13,534 (28.8)	<0.01
2	66,289 (30.7)	12782 (27.2)	<0.01
3 or more	82,484 (38.2)	17,905 (38.1)	0.84
Median income in patient’s zip code, n (%)			
$1-$38,999	61,107 (28.3)	25,659 (54.6)	<0.01
$39,000-$47,999	60,027 (27.8)	9,822 (20.9)	<0.01
$48,000-$62,999	52,038 (24.1)	7,190 (15.3)	<0.01
$63,000 or more	42,321 (19.6)	4,229 (9.0)	<0.01
Insurance, n (%)			
Medicare	126,965 (58.8)	23,263 (49.5)	<0.01
Medicaid	22,240 (10.3)	9,493 (20.2)	<0.01
Private	55,277 (25.6)	10,433 (22.2)	<0.001
Uninsured	12,307 (5.7)	3,712 (7.9)	<0.01
Hospital bed size, n (%)			
Small	40,810 (18.9)	6,015 (12.8)	<0.01
Medium	57,868 (26.8)	12829 (27.3)	0.7
Large	116,817 (54.1)	28,056 (59.7)	<0.01
Hospital region, n (%)			
Northeast	55,493 (25.7)	12,171 (25.9)	0.88
Midwest	43,888 (20.3)	7,284 (15.5)	<0.01
South	85,723 (39.7)	23,498 (50.0)	<0.01
West	30,661 (14.2)	3,947 (8.4)	<0.01
Teaching hospital, n(%)			
Non-teaching	135,386 (62.7)	20,067 (42.7)	<0.01
Teaching	80,325 (37.2)	26,881 (57.2)	<0.01
Patient outcomes			
In-hospital total foot amputations, n (%)	23,104 (10.7)	5.733 (12.2)	<0.01
In-hospital major foot amputations, n (%)	3,670 (1.7)	1,080 (2.3)	<0.01
In-hospital minor foot amputations, n (%)	19,649 (9.1)	4,699 (10.0)	0.02

### Lower extremity amputations over time

**Fig 2:**
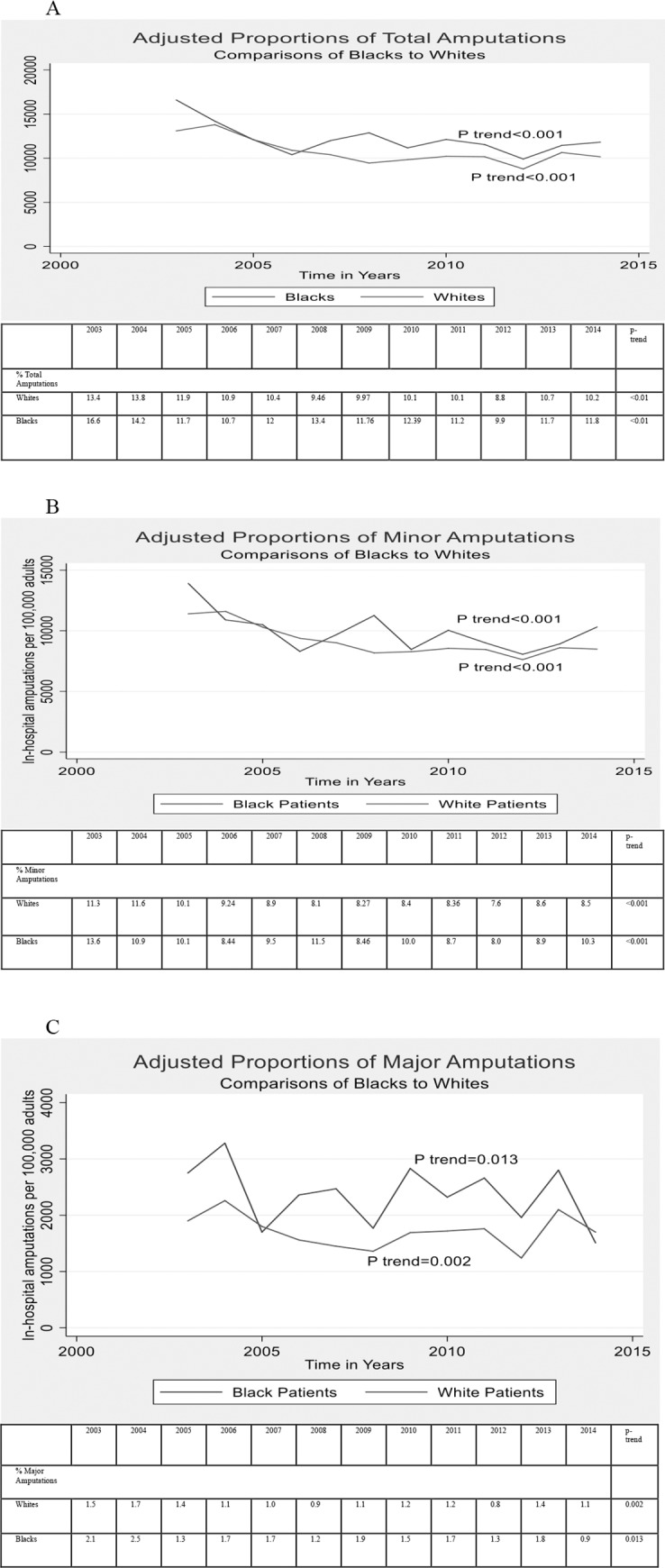
Adjusted Proportions of Amputations from 2003 to 2014 for Black and White patients; A- Total Amputations, B- Minor Amputations, C- Major Amputations.).

Fig 2 shows the time trend for and the adjusted incidence of total, minor and major amputations between 2003 and 2014. There was a significant decrease in adjusted rates of major, minor and total lower extremity amputations both among White and Black patients from 2003 to 2014. The temporal trends in major, minor and overall amputations were similar for Black and White patients (interaction terms for major amputations: p = 0.52; minor amputations: p = 0.72; and overall amputations: p = 0.96).

The proportions of major, minor and total amputations for Black and White patients for the years 2003–2004 and 2013–2014 are presented in Table 2. Table 3 shows the adjusted odds ratios for amputations between Black and White patients between 2003–2004 and 2013–2014. Black patients had 1.7-fold higher adjusted odds of major amputations in 2003–2004 compared with White patients (p<0.01). However, both groups had similar adjusted odds of major amputations in 2013–2014 (adjusted OR: 0.92 (CI: 0.58–1.44), p = 0.70). No significant differences in minor or overall amputations between Black and White patients in either 2003–2004 or 2013–2014 calendar year blocks were observed.

**Table 2:** Adjusted proportions of lower extremity amputations for Black and White patients.

	2003–2004	2003–2004	p-value	2013–2014	2013–2014	p-value
	Blacks N = 8,410	Whites N = 33,193		Blacks N = 7,731	Whites N = 40,619	
Major Amputations No. (%)	252 (3.0)	664 (2.0)	0.03	162 (2.1)	771 (1.9)	0.58
Minor Amputations No. (%)	1,043 (12.4)	3,817 (11.5)	0.3	742 (9.6)	3,414 (8.5)	0.2
Total Amputations No. (%)	1,295 (15.4)	4,447 (13.4)	0.07	897 (11.6)	4,224 (10.4)	0.17

**Table 3:** Adjusted odds ratios of lower extremity amputations for Black and White patients.

	Number of Patients	Multi-variable Un-adjusted Odds Ratio (95% CI)	p-value	Multi-variable Adjusted Odds Ratio (95% CI)	p-value
**Overall Amputations**					
**RACE (**2003–2004)					
White (ref)	33,193	1.0		1.0	
**Black**	8,410	1.2 (0.98–1.40)	0.07	1.2 (0.99–1.44)	0.05
**RACE (**2013–2014)					
White (ref)	40,619	1.0		1.0	
**Black**	7,731	1.1 (0.95–1.35)	0.16	1.1 (0.89–1.31)	0.39
**Major Amputations**					
**RACE (**2003–2004)					
White (ref)	33,193	1.0		1.0	
**Black**	8,410	1.5 (1.1–2.0)	0.013	1.7 (1.16–2.57)	0.006
**RACE (**2013–2014)					
White (ref)	40,619	1.0		1.0	
**Black**	7,731	1.1 (0.75–1.7)	0.57	0.92 (0.58–1.4)	0.70
**Minor Amputations**					
**RACE (**2003–2004)					
White (ref)	33,193	1.0		1.0	
**Black**	8,410	1.1(0.90–1.29)	0.39	1.1 (0.89–1.32)	0.41
**RACE (**2013–2014)					
White (ref)	40,619	1.0		1.0	
**Black**	7,731	1.1 (0.94–1.38)	0.18	1.1 (0.93–1.40)	0.20

### Length of hospital stay

The mean LOS in 2003–2004 was 7.6 days, (SE = 0.3) for Black patients and 6.6 days, (SE = 0.1). After adjusting for confounders, Black patients had a longer adjusted mean LOS compared with White patients (adjusted mean difference: 1.1 days (CI: 0.52 days-1.6 days; p<0.01)). The mean LOS remained longer for Black patients compared with White patients in 2013–2014, although the mean adjusted difference was smaller (0.46 day (CI: 0.18–0.73; p<0.01).

## Discussion

In the current study, we analyzed the largest publicly available inpatient care database in the United States and found that the rates of lower extremity amputations among patients with DFUs/DFIs declined from 2003 to 2014 for both Black and White patients. Not only was there a significant downtrend in rates major amputations over this time period, we found improvement in racial disparities in receipt of major amputations. While Black patients experienced higher major amputations compared with White patients in 2003–2004, no difference was seen in 2013–2014. In addition, the gaps between Black and White patients observed in earlier years for hospital length of stay narrowed.

Our results suggest that the longstanding racial disparity in major lower extremity amputations among patients with diabetic foot complications may have been recently eliminated. Previous studies using the Medicare claims database have shown that Black patients with Diabetes Mellitus have significantly high lower extremity amputation rates compared with White patients [20]. Goldberg et al used Medicare data from 1999 to 2006, and similar to our study, noted a small but significant overall downtrend in lower extremity amputation rates, the rates were greatest in high-risk (end-stage renal disease or more than three comorbidities) patients and African Americans had higher rates of amputation in both the high-**risk** and low-**risk** groups [20]. In another Medicare claims-based study by Newhall et al, patients with Diabetes Mellitus and concurrent peripheral vascular disease followed from 2007 to 2011 found Black patients had higher rates of major lower extremity amputations compared with other races [21]. In contrast to these two studies, we found that the racial disparity in major amputation rates among patients with Diabetes Mellitus and DFUs/DFIs decreased and ultimately disappeared by 2013–2014. Several differences between the NIS and the Medicare claims database can potentially explain the discordant results obtained. First, using NIS, we were able to include adult patients of all ages, as opposed to the Medicare database that only include patients 65 years or older. Second, we could study patients with all types of insurance providers, including patients who are uninsured. The Medicare database includes patients insured by Medicare exclusively. Third, our follow-up period was from 2003 to 2014, while the other two studies had shorter follow-up periods ending in 2006 and 2011, respectively. It is important to note that we also found a non-sustained decline in foot amputations between 2003 and 2006 for Black patients (where when compared to White patients, in the year 2005 Black patients had lower occurrences major foot amputations, and in 2006 Black patients had lower overall and minor foot amputations. Given that this study was observational/descriptive the etiology for this finding is not entirely clear.

Although our study was not designed to uncover the reason for the observed resolution of the racial disparity in lower extremity major amputations, several hypotheses are plausible. One possibility may be the expansion of healthcare coverage for uninsured individuals as a result of the Affordable Care Act (ACA). Our study found that the proportion of uninsured Black patients dropped between 2003 and 2014. Loehrer et al found that the racial disparities between Black and White patients in severity of peripheral vascular disease was eliminated in the state of Massachusetts following implementation of the healthcare reform that served as the foundation for the ACA [22]. Research has shown that having insurance increases access to healthcare [23], which in turn improves treatment outcomes [24]. Along the same lines, a study using the Kaiser Permanente database looked at racial differences in lower extremity amputations between Black and White patients with Diabetes Mellitus from 1995 to 1998. No differences in amputation rates were identified based on race [25]. All patients had access to healthcare. Our larger study on a broader national scale supports these results.

Aside from access to healthcare, it is also possible that improvements in racial disparities in surgical outcomes may be directly correlated to improved surgical practices. For example, Mehtsun et al., using the Medicare inpatient claims database from 2005 to 2014, found significant improvement in racial disparities in post-operative mortality between Black and White patients [26]. The authors suggested that improvement in general surgical care over years might be the driving force for the observed results. Also, the Centers for Disease Control and Prevention noted that better care in outpatient glycemic control may also play a role in the observed decline in foot amputations [27]. Thus, the decrease in overall amputations we report may also be supported by non-invasive interventions and preventive measures that may lead to less amputations.

Despite similar rates of major amputations among Black and White patients in 2013–2014, Black patients had a persistently longer LOS compared with White patients over the years. Similar findings on LOS have been reported in other studies following other surgical interventions [28,29]. Based on our results, one possible hypothesis that can explain this finding is insurance status. We found that Black patients were more likely to be insured over time, but were still more likely to be uninsured compared with White patients. Lack of insurance may have contributed to longer LOS for multiple reasons. First, more therapeutic interventions that otherwise could have been offered in the outpatient setting may have been performed before discharge due to less certain compliance with follow-up appointments. Also, appropriate arrangement for timely post-discharge follow-up assessments, and placement in subacute rehabilitation facilities after discharge could have been more challenging for patients without insurance.

There are some limitations to this study that should be noted. First, the NIS is an administrative database that uses ICD-9 and ICD-10 CM codes, and thus is prone to incorrect or missing codes [30]. However, the codes we chose have been used previously to study lower extremity amputations in the setting of Diabetes Mellitus and DFUs/DFIs [31,32]. Second, we recognize that race is a self reported social construct that is tightly linked to ethnicity and reflects historic and persisting socio-cultural dynamics of the country. The Office of Management and Budget standards format for reporting race/ethnicity would classify each person with both a race and ethnicity. Some databases capture both, while others capture only one or the other. NIS only captures data on race (not ethnicity), which may limit direct comparisons with other national datasets such as the National Health and Nutrition Examination Survey. Third, the NIS does not collect laboratory, imaging or medications administered during patients’ hospital stay. Therefore, we could not include this information as part of our resource utilization analysis. Also, the median income in the patient’s zip code has been used as a surrogate for the patient’s household income. Finally, though we used a widely used index to measure patient’s comorbidity, the Charslon comorbidity index, it is possible that at least some were missed.

The current study also has multiple strengths. The main strength is that it uses the largest publicly available all payer inpatient database in the United States, minimizing the likelihood of a beta error. Most importantly, NIS is nationally representative because it includes patients from hospitals small, medium and large, teaching and non-teaching, rural and urban, privately or publicly owned, for profit and not for profit, across 45 states, including burn centers. Therefore, our results should be reflective of all patients admitted to a hospital with DM and DFI/DFU across the United States [33].

In conclusion, lower extremity amputations for both Black and White patients significantly decreased between the years 2003 and 2014, and disparities in major amputations appear now appear have been eliminated. This may suggest greater adherence to guideline directed therapy and practices for diabetic foot complications irrespective of patient’s race and/or increased access to care may have contributed. Future studies should target identifying specific contributors for this outcome with focus on sustainability. Opportunities for improvement in the racial disparity between White and Black patients still exists in this setting, especially in healthcare resource utilization/length of stay. In an era where Healthcare delivery is increasingly measured by indicators of quality and efficiency, exploring the reasons behind this persistent disparity in order to address it are more relevant than ever before.
